# Supplementary material for: Exploring the structural landscape of DNA maintenance proteins
Source: Nat Commun. 2024 Sep 5;15:7748. doi: 10.1038/s41467-024-49983-7 (PMC11377751; doi:10.1038/s41467-024-49983-7)
Supplement: Supplementary file 1 — Supplementary Information [file 41467_2024_49983_MOESM1_ESM.pdf]

Supplementary Information

Exploring the structural landscape of DNA maintenance proteins

Kenneth Bødkter Schou[1,2,3*], Samuel Mandacaru[2], Muhammad Tahir[2], Nikola Tom[4], Ann-Sofie Nilsson[3], Jens S. Andersen[2], Matteo Tiberti[5], Elena Papaleo[5,6] & Jiri Bartek[1,3*]

1. Genome Integrity, Danish Cancer Institute, Danish Cancer Society, Strandboulevarden 49, 2100 Copenhagen, Denmark.
2. Department of Biochemistry and Molecular Biology, University of Southern Denmark, Campusvej 55, 5230 Odense M, Denmark.
3. Division of Genome Biology, Department of Medical Biochemistry and Biophysics, Science for Laboratory, Karolinska Institute, Solna 171 77, Sweden.
4. Lipidomics Core Facility, Danish Cancer Institute (DCI), DK-2100 Copenhagen, Denmark
5. Cancer Structural Biology, Danish Cancer Society Research Center, Strandboulevarden 49, 2100 Copenhagen, Denmark.
6. Cancer Systems Biology, Section for Bioinformatics, Department of Health and Technology, Technical University of Denmark, 2800 Lyngby, Denmark

*To whom correspondence should be addressed. Jiri Bartek Tel: +45 28 99 01 59; E-mail:

jb@cancer.dk; Kenneth Bødtker Schou E-mail: kensch@cancer.dk

Supplementary Figure 1

a

New domains in human genome maintenance proteins

New domain	Proteins
BRCT	SMARCC1, SMARCC2, TERB2
OB fold	FAM35A (SHLD2/RINN2), POLE2, TDRD3, SPATA22, C17ORF53 (HROB), SPIDR
KU70/KU86	M1AP, INTS14
PARP	TEX15 (isoform 2), TASOR, TASOR2
Myb/SANT	TIMELESS, SMARCC1, SMARCC2
UBA	ZC3H12D, RAP1, N4BP1, UBXN2B
SAP	PARP1
TUDOR	SETDB1 (the third 180-260), PWWP3A, BRMS1L , SUDS3, KIF2C, KIF2A
WSD	RSF1, CECR2
BAH	GATAD1, ARID5B
Alba_2	Schlafen
HEAT/ARM repeats	TERB1, INTS7, INTS5, INTS4 INTS2, INTS1
TPRs	ESPL1, INTS8, INTS10
RFC1_CT	CHTF18
DSPn	PARD3
SFI1	CCDC191, C1ORF167, POC5
NNCH/NDC80	HAUS3, HAUS6, HAUS7, CEP44, TEDC1
NDC10	KIAA1958, QRICH, KCTD1 (isoform b), ZMYM2. ZMYM3, SMYM4

b

New human genome maintenance candidates

New domain	Known domain(s)
Myb/SANT	CRAMP1, LOC100506514 (pseudogene)
ERCC4	C1ORF146, C9ORF84 (SHOC1)
PARG Macro	AKAP3, AKAP4, AKAP11, SPHKAP
UVR	TRANK1
FLAP exonuclease	FAM120A FAM120B, FAM120C, ASTE-1
DDE endonuclease	GVQW3/FLJ37770, C21ORF140,
MIS18	FAM72A, FAM72B, FAM72C, FAM72D
TUDOR	ZNF395, RPL26L1,BCMS1L
Alba_2	SAMD9L
POLO box	C5ORF34
SLF2	FAM178B
SAP	LETM1, LETM2, LETMD1, CDNF, NAB2, NAB1, MANF, SLTM, C2orf49, Emerin, RNF103, RNF34, RFFL, DPPA4, HMGXB3 LAP2B, ANKLE2, KLHL2
Metallo-beta-lactamase	MAP1A, MAP1B, MAP1S
Beta-TrCP D domain	C14ORF119, SAMD4A/SMAUG1, ZCCHC2, ECT2L,
WSD	KIAA2026
YEATS	RNF166 (isoform 4), SMARCD3, SMARCD2, SMARCD1

Supplementary Figure 1.

Candidate domains in human genome maintenance proteins. **a**, List of proteins in GM processes with predicted domains as identified from the profile-HMM survey. **b**, Human genome maintenance candidates. List of proteins predicted to have roles in GM processes inferred from the presence of one or more GM telltale domains.

Supplementary Figure 2

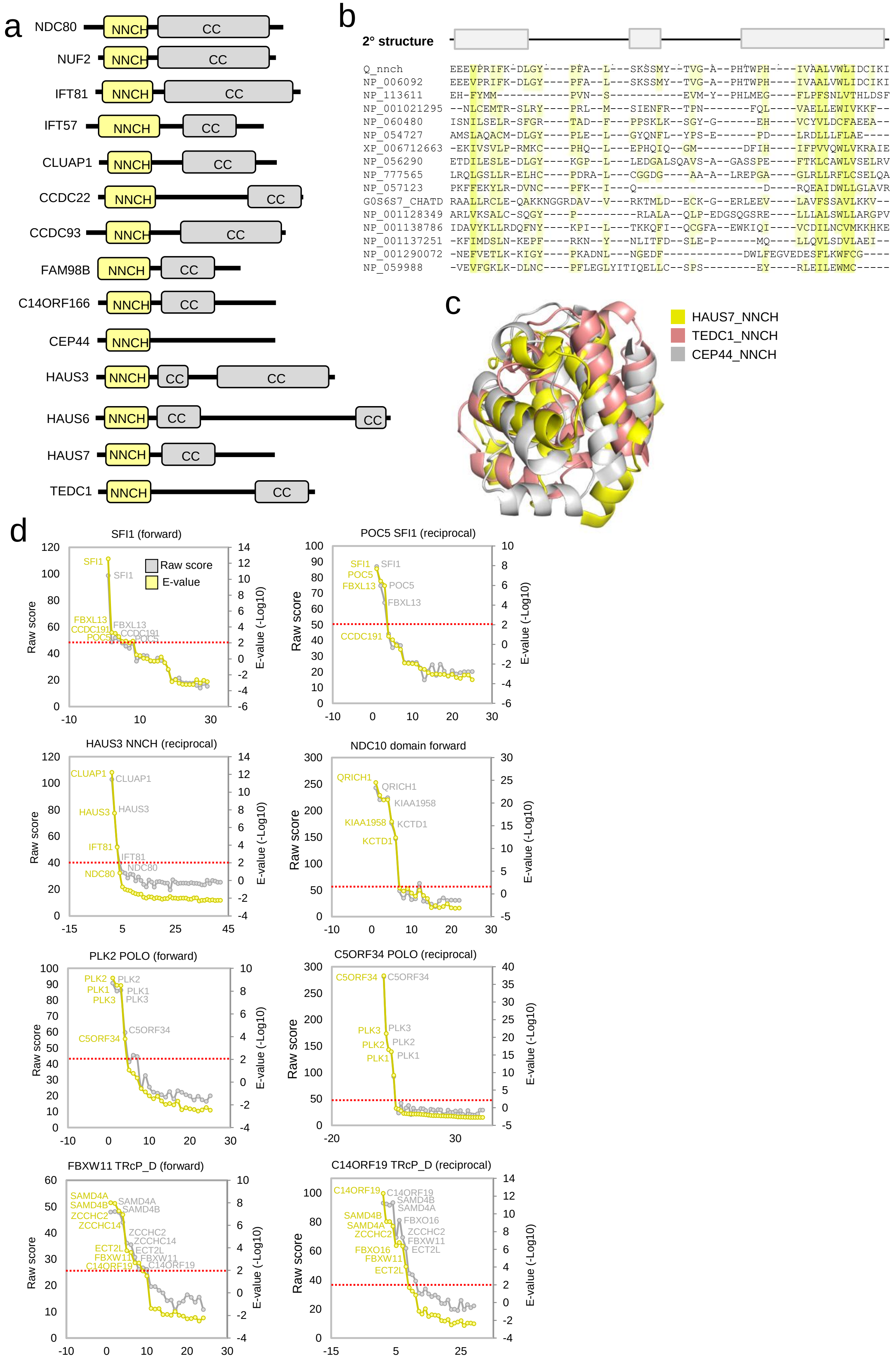

Supplementary Figure 2.

Extended NNCH domain family in humans. **a**, Family of human NNCH proteins. **b**, MSA of NNCH domain sequences. Conserved residues shown in yellow were assessed using the Clustal W method with modifications. Predicted secondary structures are shown above the MSA. Boxes indicates alpha-helices. **c**, AlphaFold2 prediction of NNCH domains. NNCH domains were superimposed in Pymol. **d**, Probability plots of profile-HMM remote homology searches using either example of human protein structures found in proteins tied to mitotic functions. Source data are provided as a Source Data file.

Supplementary Figure 3

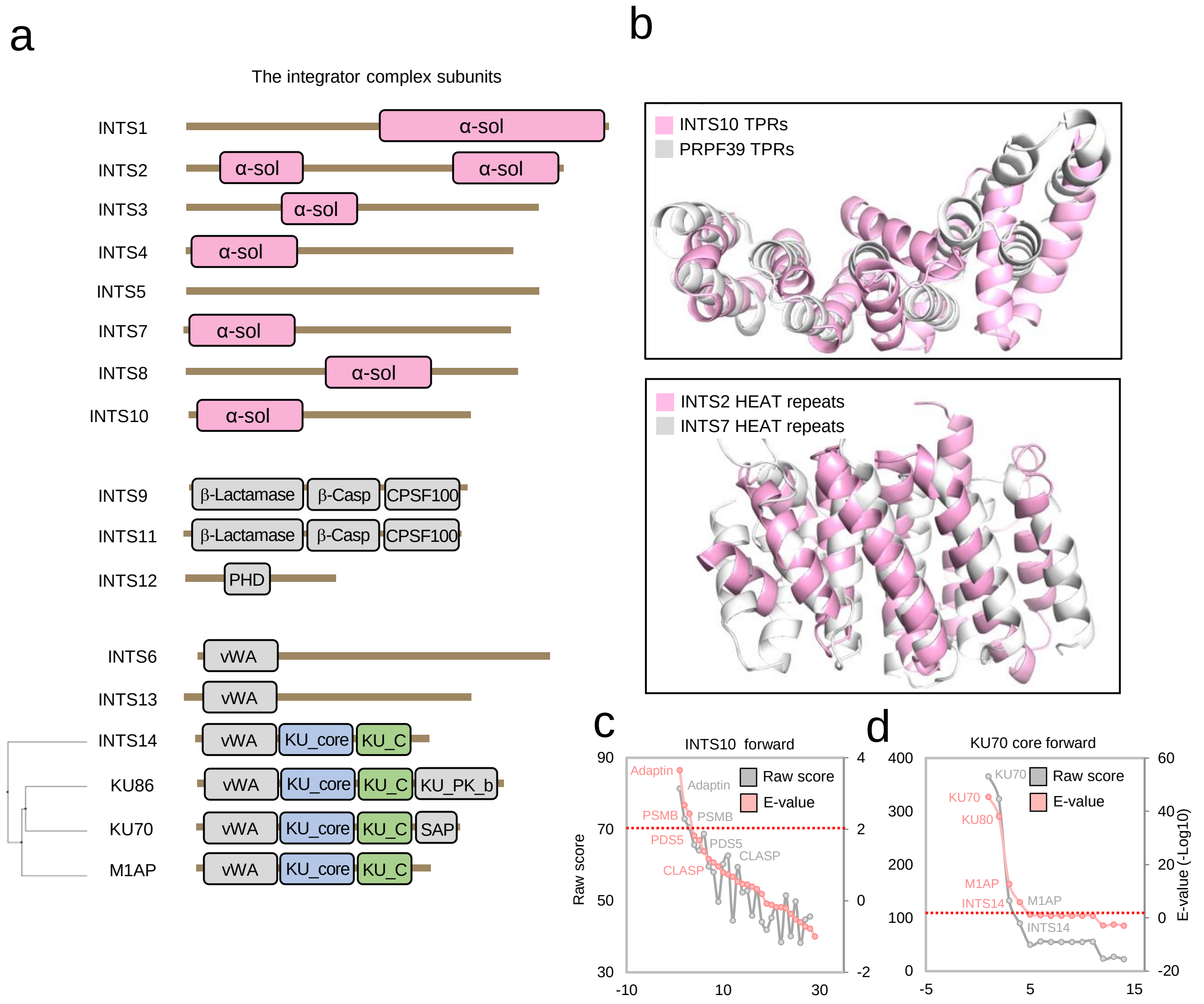

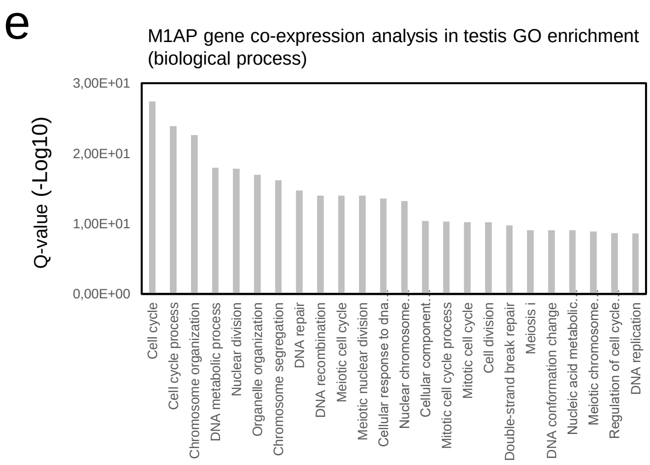

f

M1AP gene co-expression analysis in testis

Gene	PCC
FANCI	0.85
DPEP3	0.84
MSH2	0.84
SMC1B	0.84
ANKRD31	0.83
KIF11	0.83
TOPAZ1	0.83
SYCP3	0.83
CENPH	0.83
CHEK1	0.82
KDM1A	0.82
RAD9B	0.82
CEP85	0.82
GINS2	0.82
CSE1L	0.82
BRCA1	0.82
DMC1	0.82
TCL1A	0.82
FEN1	0.81
MAD2L1	0.81
CCDC79	0.81
TDRD12	0.81
SHCBP1	0.81
CASC5	0.81
HSPA14	0.80
PNMA5	0.80
SPO11	0.80
ASZ1	0.80

Supplementary Figure 3.

Conserved structures of the integrator complex members and the M1AP protein**. a**, Schematic representation of the human family of integrator (INT) protein structures as well as the INTS14 paralogs KU70, KU86, and M1AP. The similar HEAT, ARM, and TPR arrays are here collectively designated α-solenoids (α-sol). **b**, INTS2 and INTS10 AlphaFold 3D models showing the arrays of TPRs comparable to those of PRPF39 and INTS7, respectively. **c**, Probability plots of profile-HMM remote homology searches using human INTS10 as a query (forward search). **d**, Probability plots of profile-HMM remote homology searches using the human KU_core domain of KU70as a search query (forward search). See Fig. 1f for the KU_core reciprocal search. **e**, M1AP gene co-expression and GO enrichment analysis using RNA seq patient data from testis available in the GTEx database. M1AP co-expressed genes are ranked according to pearson correlation coefficients (PCC) as shown. **f**, Top co-expressed genes of M1AP in testis. Source data are provided as a Source Data file.

Supplementary Figure 4

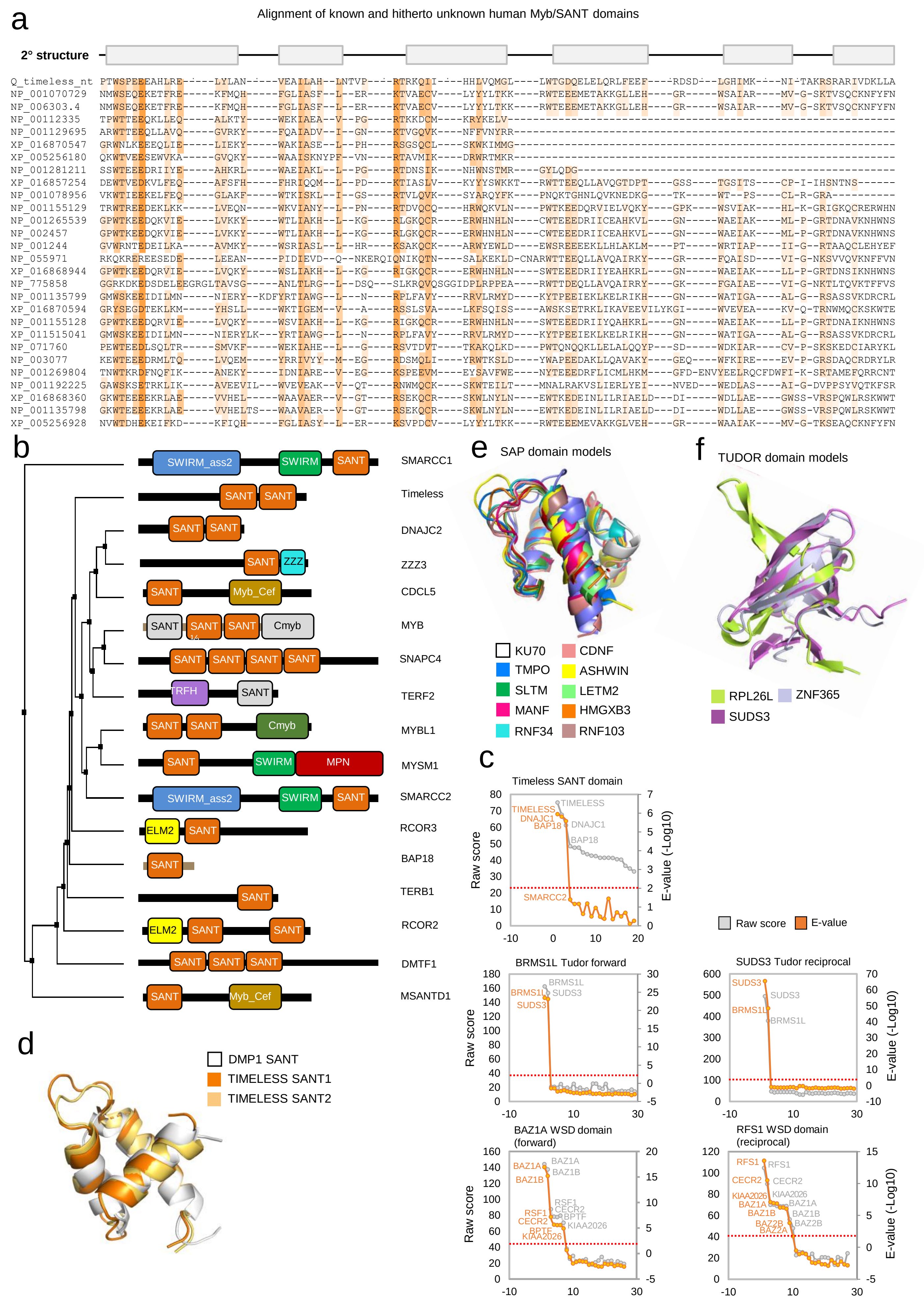

Supplementary Figure 4.

Examples of DNA and chromatin-binding structures identified in the survey. **a**, MSA of SANT domain-containing proteins in humans. Conserved residues shown in orange were calculated using the Clustal W algorithm. Predicted secondary structures are shown above the MSA. Boxes indicates alpha-helices. **b**, Schematic representation of the human family of SANT domain family of proteins. c, Probability plots of profile-HMM remote homology searches using the predicted human Timeless SANT, BRMS1L and SUDS3 TUDOR, or BAZ1A and RFS1 WSD domainsas queries (either forward or reciprocal searches as indicated). **d**, Structures of the predicted SANT domains in Timeless superimposed with the SANT domain in DMP1. Superimposition was performed in PyMol. **e**, Structures of SAP domains as predicted by AlphaFold. For comparison the solved SAP domain of human KU70 is shown. SAP domains were superimposed in PyMol. **f**, Structures of TUDOR domains as predicted by AlphaFold. The TUDOR domains were superimposed in PyMol. Source data are provided as a Source Data file.

Supplementary Figure 5

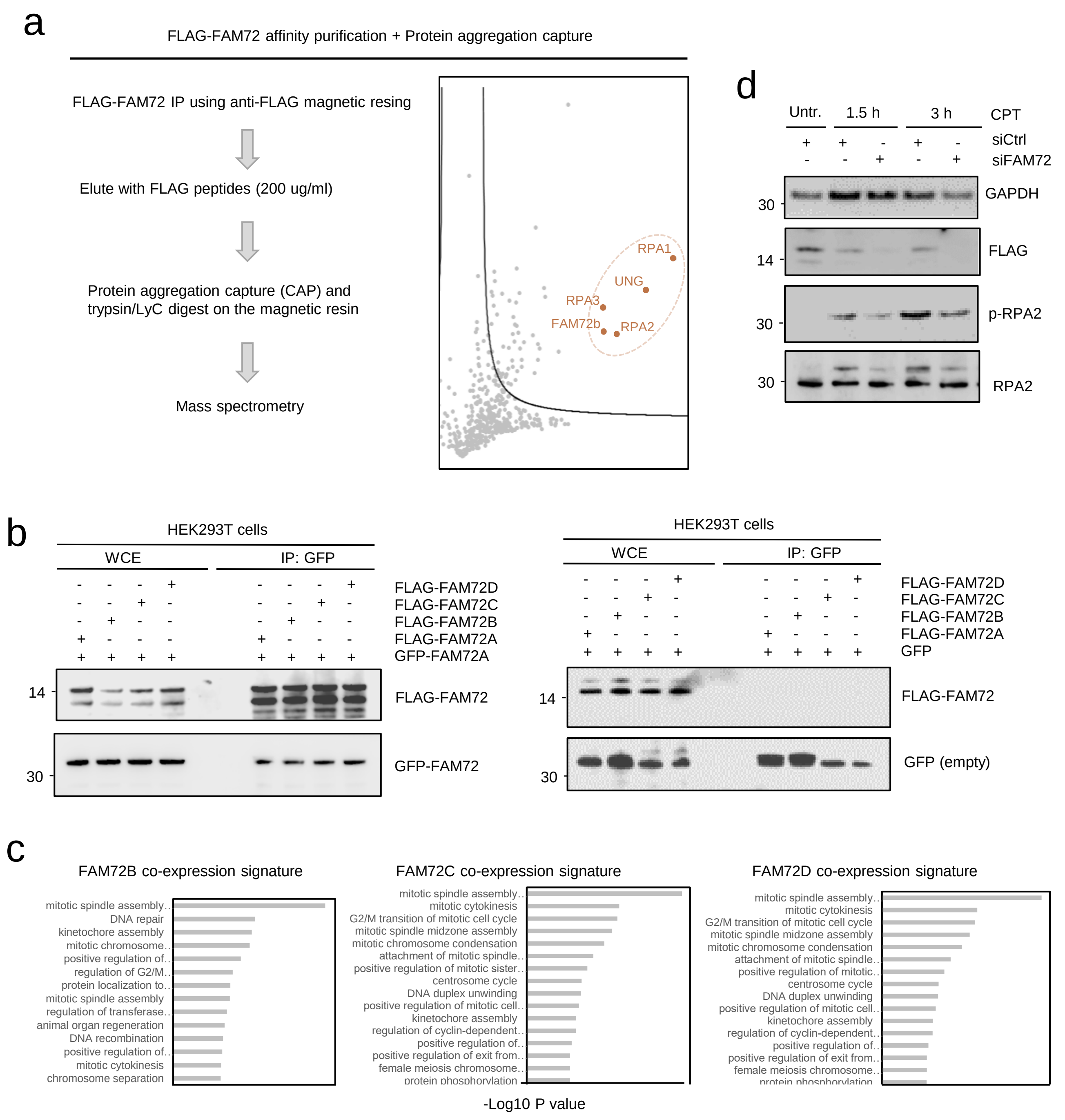

Supplementary Figure 5.

Assessment of functional properties the FAM72 family proteins. **a**, Protocol for FLAG-FAM72B IP, elution, protein aggregation capture, protein digest, and mass spectrometry. **b**, Immunoblots of eluted proteins from GFP immunoprecipitation assays of extracts from HEK293T cells co-expressing either GFP (empty) together with FLAG-FAM72A-D or GFP-FAM72A together with FLAG-FAM72A-D. Proteins were probed with the indicated antibodies. WCE = sample processing control. **c**, Gene co-expression and GO enrichment analysis results for individual FAM72B, FAM72C, or FAM72D genes. **d**, Immunoblot of cell extracts from FLAG-FAM72B-expressing HEK293T cells after silencing FAM72B with siRNA for three days followed by exposure to CPT the indicated times. Immunoblots are representative results of two individual experiments (X=2). Source data are provided as a Source Data file.

Supplementary Figure 6

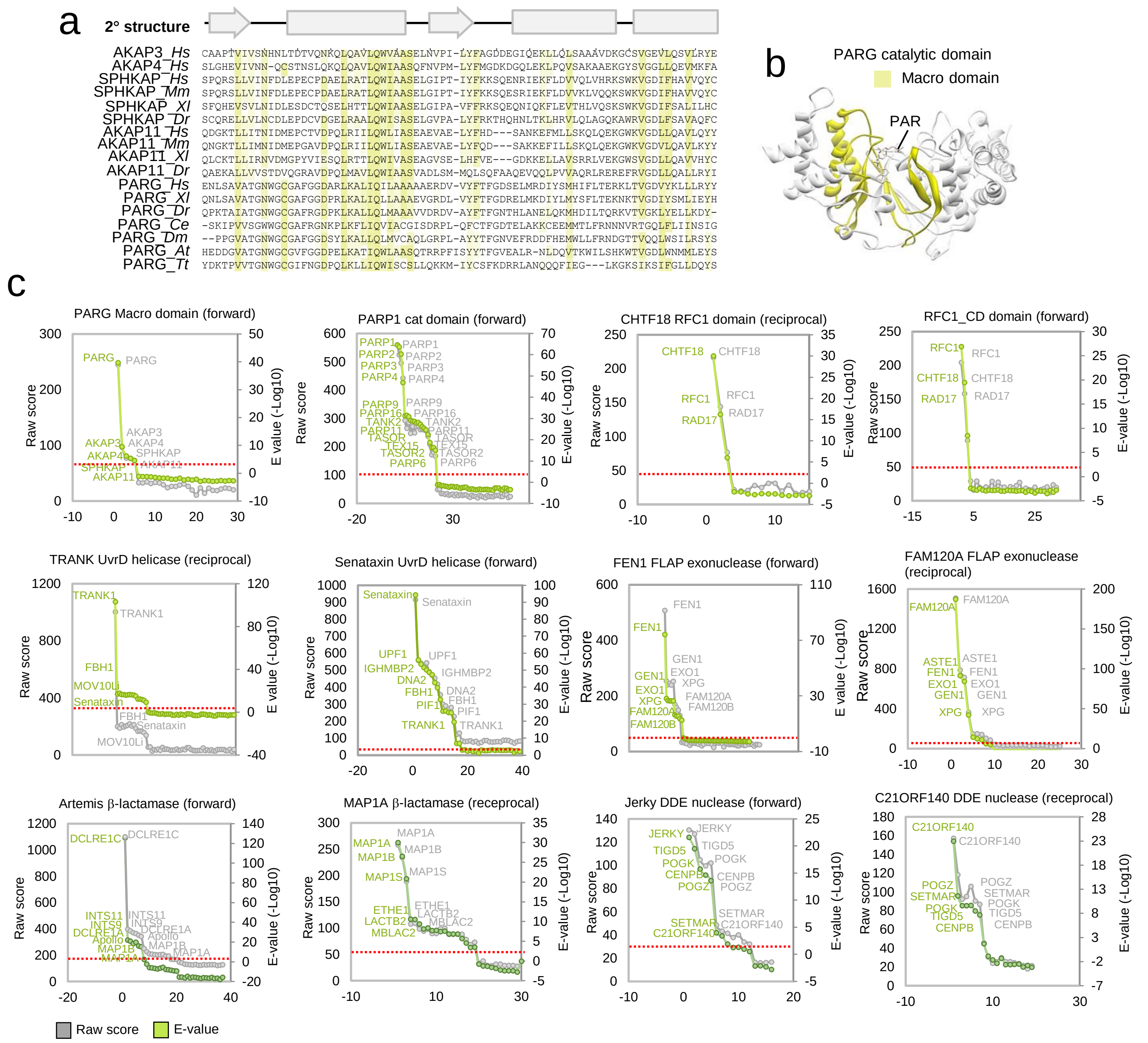

Supplementary Figure 6.

Examples of identified GM proteins with putative catalytic functions. **a**, MSA of PARG-type Macro domain-containing proteins across species. Conserved residues shown in yellow were assessed using the Clustal W algorithm. Predicted secondary structures are shown above the MSA. Boxes indicates alpha-helices and arrows indicate beta-sheets. **b**, PARG domain of PARG (PDB: 3SIG) showing the portion with homology to AKAP protein family members highlighted in yellow. This domain represents the Macro domain here shown bound to a PAR unit. **c**, Probability plots of profile-HMM remote homology searches using established (forward searches) GM nuclease domains and predicted GM candidates (reciprocal searches) as search queries. Source data are provided as a Source Data file.

Supplementary Figure 7

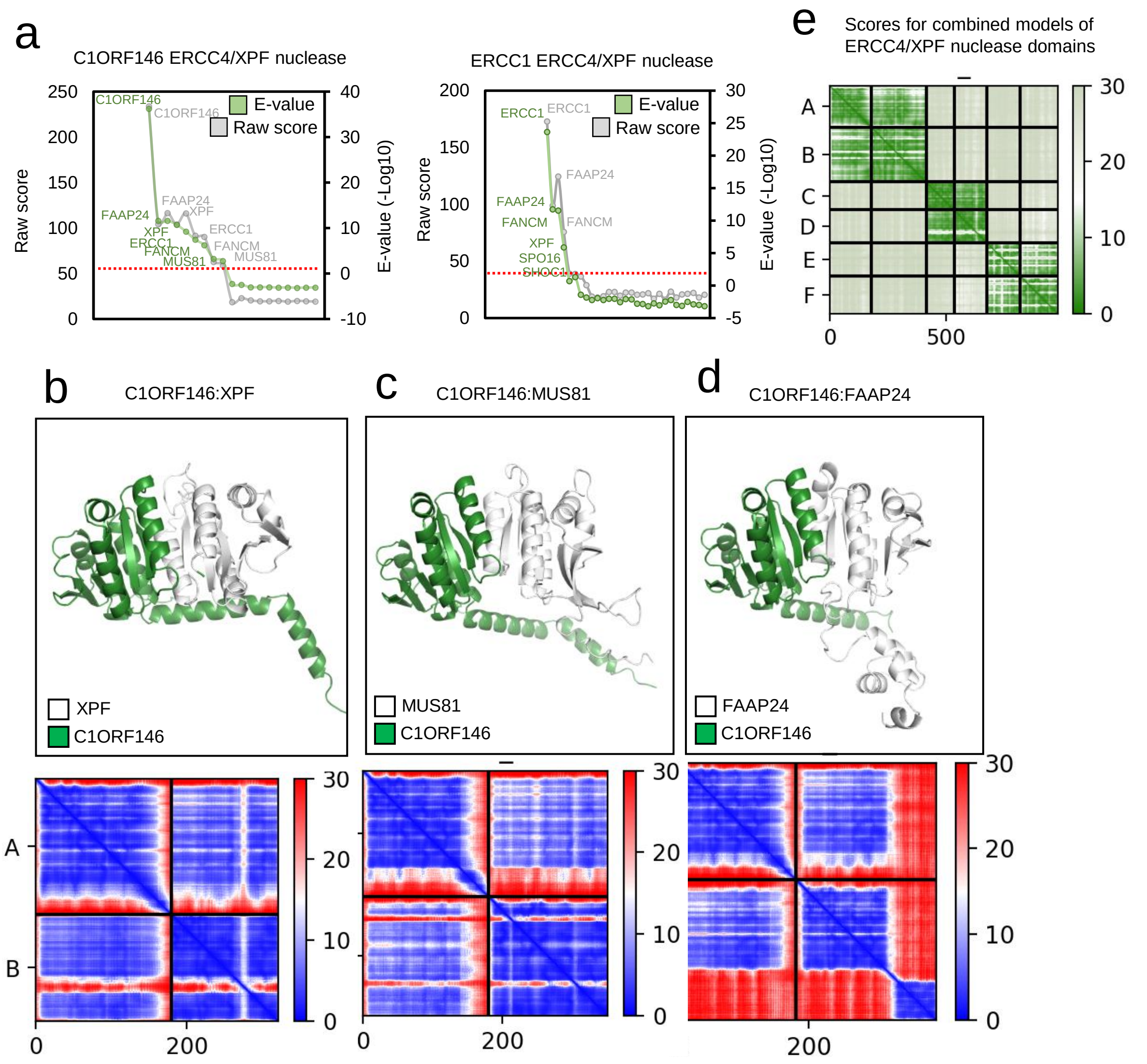

Supplementary Figure 7.

Assessment of the C1ORF146 protein. **a,** Probability plots of profile-HMM remote homology searches using either the predicted human ERCC4 domain of C1ORF146 a search query (reciprocal search) or the ERCC1 ERCC4 domain (forward search) as search queries. **b-d,** Hypothetical ERCC4 heterodimer complexes between C1ORF146 and either XPF, MUS81, or FAAP24 as predicted by ColabFold104. Below the predicted complexes are shown the predicted alignment error (PAE) for ERCC4 heterodimer complexes C1ORF146-SHOC1, ERCC1-XPF, and MUS81-EME1. e, PAE Scores for combined models of ERCC4/XPF nuclease domains. Source data are provided as a Source Data file.
