# Supplementary material for: Exploring the structural landscape of DNA maintenance proteins
Source: Nat Commun. 2024 Sep 5;15:7748. doi: 10.1038/s41467-024-49983-7 (PMC11377751; doi:10.1038/s41467-024-49983-7)
Supplement: Supplementary file 3 — Description of Additional Supplementary Files [file 41467_2024_49983_MOESM3_ESM.pdf]

## Description of Additional Supplementary Files

File Name: Supplementary Data 1
Description: MSA of SMARCC1 and 2 BRCT domains aligned with BRCT domains from other proteins and across species. Conserved residues highlighted by colors were assessed using the Clustal W algorithm.

File Name: Supplementary Data 2
Description: Expanded MSA of SPIDR OB fold domains aligned with OB fold domains from other proteins and across species. Conserved residues highlighted by colors were assessed using the Clustal W algorithm.

File Name: Supplementary Data 3
Description: Expanded MSA of M1AP KU_core domain aligned with KU_core domains from KU proteins from humans and other species. Conserved residues highlighted by colors were assessed using the Clustal W algorithm.
