# Supplementary material for: Exploring the structural landscape of DNA maintenance proteins
Source: Nat Commun. 2024 Sep 5;15:7748. doi: 10.1038/s41467-024-49983-7 (PMC11377751; doi:10.1038/s41467-024-49983-7)
Supplement: Supplementary file 4 — Supplementary Data 1 [file 41467_2024_49983_MOESM4_ESM.pdf]

Supplementary Data 1

SMARCC2 BRCT domain alignment

```
Q_smarcc1/1-57         NNCLT---RP-NIYL--I-P--D-I-NKLKDIIKRHQG-T--FT---K-A---S-H---HIY-----PWKVHVKWILDTDIF--E-WM---DY
NP_524373.1/1-57       ADLYR---IP-YIYI--R-P--E-I-GKLREILDNRRV-E--IV---E-A---T-H---IVY-----RWRVSASWIVDLEQY--E-WM---DY
NP_729947.2/1-56       MRLMR---GI-RVAI--T-N----V-RRLYAMLTYHGA-V--VT---F-N---T-H---LVC-----IIIVTPDWVTDSLKY--N-CM---TY
NP_001077856.1/1-56    SKLME---GV-VFVL--S-G----F-STLRSQALTMGA-T--YQ---W-S---T-L---LIC-----GTIISKEWITECYAQ--K-LV---QY
NP_001322042.1/1-53    ---ME---NV-VATV--S-G----Y-FKLIKLISHSGA-S--YV---M-I---T-H---LVC-----TVVVNHRWVEECVKE--R-RV---PY
NP_572217.1/1-56       NQLLK---GV-VLVI--S-G----I-ADLRSKAVALGA-K--YK---W-C---T-H---LIC-----GKIVTRSWIEKCYEL--K-YL---RY
NP_189979.3/1-60       DGPFS---GL-IICV--T-G----L-KQVKEATERLGG-E--YS---L-C---T-H---LVV-RRETLYIVTLGWFVDSVCR--V-KL---FY
NP_006288.2/1-56       GKILQ---GV-VVVL--S-G----F-SELRDKALELGA-K--YR---W-S---T-H---LIC-----GRIVRKEWVLDCHRM--R-RL---RY
XP_016871577.1/1-58    EEWLS---SL-RAHV--V-RT-G-I-ELFEKQIVQHGG-Q--LC---Q-V---T-H---IVV-----AQLVKSAWLSLCLQE--R-LV---GF
NP_001318917.1/1-57    PAFTK---EL-VLCG--S-A----L-KLMESLAVRFNA-T--IS---W-V---T-H---VIAS----KWIINAAWMKASLKA--Q-PV---PF
NP_192222.4/1-57       RRNLA---HV-RVLF--S-Q--N-L-KQQKKIMVRLGI-S--PA---S-S---T-H---FIA-----KFVVTPIWLESCAQT--C-LI---SY
NP_188785.2/1-57       RRDLG---SI-CVLF--S-Q--H-L-KHQKKILARFDI-S--EA---M-A---T-H---FIA-----KPVVTTQWLESIDQV--I-YV---MY
NP_001118498.1/1-57    RPNGQ---HY-RILL--M-D--I-C-AWLTEVIRKLGG-T--VT---G-S---T-H---IVT-----AWIVSPSWLKESVRE--R-FA---SH
XP_016881567.1/1-58    SKVLA---DV-AIIF--S-G----LHTREHYHATALGA-K--IL---LVA---T-H---LIA-----LHVVNPDWLWSCLER--D-KV---LF
NP_006288.2/1-59       PDFFQ---GK-HFFL--Y-G--E-F-RKLIRYVTAFNG-E--LE---M-V---Q-F---VIT-PS--LAFVRPRWIYSCNEK--K-LL---LY
NP_729947.2/1-52       ---LE---RY-IITS--E-G----F-VRLQQMAEECGA-I--YT---L-N---T-V---VVC-----IPMVNALWLSDVCI---N-LS---QY
XP_011533060.1/1-57    RGKKP---TR-TLVM--T-S----M-NVVIQVVDKLKGFS--IA---V-T---T-H---VLS-----CWVLSYDWVLWSLEL--H-WI---PF
NP_725088.1/1-56       PELLK---DI-VVYV--E-V----R-EGVKSIIAKMGA-Q--VK---L-T---T-H---VVF-----IPVVSILWIEACKVQ--K-IC---QF
XP_005247233.1/1-55    -PPFQ---DC-ILSF--L-G----F-TNMEEMTEMQGG-K--YL---G-C---T-H---LVV-----LYVVKQEWFWGSIQM--A-RA---MY
XP_011514285.1/1-56    LTPEL---TP-FVLF--T-G----F-QQYIKKLYILGG-E--VA---A-C---T-H---LIA-----KHIVTPEWLEECFRC--K-FI---NY
NP_193839.4/1-57       HGFSK---KL-VLSC--S-G----L-TVIAEFAELSGV-T--IS---W-V---T-H---VIAS----KWLTIDWIKACMKN--K-YV---PY
NP_612047.1/1-56       SDLFQ---GI-SIFV--N-G----R-DELKRIMMVHGG-T--FH---E-T---T-Y---IIA-----SKFISAKWVVDCLEK--K-IV---PY
NP_001308971.1/1-58    APILK---DV-VAYV--E-V--W-SSKTFTTQLVDMGA-K--VS---F-V---T-H---VIF-----VKLVSVLWVEKCRTA--A-HI---LF
NP_009229.2/1-56       ERVNK---RM-SMVV--S-G----L-MLVYKFARKHHI-T--LT---I-T---T-H---VVM-----KWVVSYFWVTQSIKE--K-ML---DF
NP_729306.1/1-54       -KAFE---GQ-KICF--F-G----F-QHMVDVLLENGG-V--CA---D-C---S-H---VVV-----THILKSDWFWYTIQN--Y--A---DY
NP_729947.2/1-52       ----K---PP-KVIF--S-Q----V-EALKKAVLILGG-I--VV---P-A---T-H---LVM-----DYVLKSSWIADSAKA--K-FV---PY
NP_001327947.1/1-55    --QEH---EP-KFFI--V-S----G-NEYQQIIRRLKG-K--CC---SHA---T-H---FIA-----SWILKTDYVADSKEA--K-LL---PY
NP_177856.2/1-55       -PGFE---SL-CICS--S-Q----H-ELLRNLSVVLGA-D--FV---L-V---T-H---LIC-----IISVTPDWLYECVRQ--Q-VV---NF
NP_611934.1/1-57       SEVLR---GK-NLVF--S-G----LVSRAYFIAKSLGA-E--VK---I-I---T-H---LVA-----IKVVNANWLWTCAER--E-HV---LF
XP_011513589.1/1-58    STRFP---GV-AIYL--V-EP-R-M-AFLTGLARSKGF-R--VL---C-A---T-H---VVM-----PALLDISWLTESLGA--Q-PV---CR
NP_001327092.1/1-57    DLALS---GY-SVFI--D-A--D-I-RQVSQVAVEGGA-K--LM---I-A---S-H---VVC-----SNLVTPLWLQKTLEE--P-TQ---QM
XP_011510659.1/1-56    NMVMS---DV-TISC--T-S----L-EEVHKYVQMMGG-R--VY---L-V---T-H---LIA-----KPILLPSWIKTLWEK--Q-EK---RY
XP_016860102.1/1-56    QRRDG---PL-VLIG--S-G----L-KMLSELAVILKA-K--KY---F-V---T-H---VVV-----CWILKFEWVKACLRR--V-CE---KY
NP_568851.2/1-57       TSIFS---NM-IFYF--V-N--V-P-ETFHKMVVENGG-K--FS---L-V---T-H---CIA-----RDVIHFSWVLDCCSR--K-ML---PK
NP_001119373.1/1-56    KLIFQ---GV-SIFV--D-G----F-QELKGYMMKYGG-R--FE---F-V---T-H---IIC-----LPVVKPTWIVDSISA--R-LL---PY
XP_016861126.1/1-53    ----K---QY-IFQL--S-S----L-IDYCHLIEKLGG-L--VIE--F-C---T-H---IVV-----KWVLHRSYLEACRTA--H-FV---DY
NP_001163122.1/1-58    GKSRT---RI-RTLV--H-T--N-M-NVIRKALRKLRGMR--LD---V-T---T-H---LVS-----VWIVSYQWVLASIRA--K-WI---PY
XP_011514285.1/1-57    SQGEV---GK-GACP--ADP----V-SALWALVTFYGG-D--CQ---L-C---T-H---LIV-----IKIVTPDWVLDCVSE--T-KK---FY
NP_001078764.1/1-55    --ILK---GC-KIVF--S-R----VFHPLWKMAEELGA-T--CA---V-V---T-H---VVA-----KYVVHRGWIDAANYL--M-KQ---NF
NP_001032961.1/1-56    STIFS---GV-AIYV--N-G----Y-EELRKLMMLHGG-Q--YH---Y-T---T-H---IIA-----EKVIRPEWIVESIKA--R-LL---PY
NP_001325202.1/1-55    --ILA---GC-RIVF--S-R----IIHPLWQTAEQFGA-V--CT---V-V---T-H---VVT-----RFVVHPGWVEASAFL--Q-RA---LY
XP_016861323.1/1-57    CTSMM---NL-VLCF--T-G--F-R-VRLVTLVHHMGG-V--IR---F-V---T-H---LVA-----TPIMKPEWIYKAWER--N-EQ---AA
XP_011510659.1/1-56    PKPLH---KV-VVCV--S-K----K-SELNGIAASLGA-D--YR---F-V---T-H---FIY-----VHIVSEHWLLDCAQE--K-HL---LY
NP_192120.4/1-56       PKTYS---GV-KFAL--V-G----F-NSLRSKLVSGGG-V--DV---F-C---T-H---LIV-----KVVVTGSWVDHSFDI--M-LD---SI
XP_016867011.1/1-55    NQEST---AP-KVLF--T-G--V-V---GERAVLALGG-S--LA---A-A---S-H---LVT-----IPILSLDWLHQSRKA--F-FL---EY
XP_011510659.1/1-54    --LLD---GC-RIYL--C-G----F-DKLRRLINSGGG-V--RF---L-V---T-H---VIV-----PHVVGAKWLLECFSK--Y-ML---PY
NP_524909.1/1-56       STAMR---DL-QVSA--T-G----I-EELSRLINWMGG-I--YF---F-T---T-H---LIS-----VPVMHVDWVQYVWDQ--R-RS---GI
NP_006428.2/1-57      -GIFA---NC-IFCL--K-V--KYL-KKLQTDIKENGG-K--FS---L-C---T-H---IIL-----VHIANPDFIWKSIRE--R-LL---NY
NP_001332576.1/1-55    -SCLL---SC-CVYF--Y-P----Y-KRLMLEVLMAGG-K--VS---L-A---S-H---LVV-----LHVVSSHWLEESLQR--E-KL---VY
NP_177856.2/1-55       --PFS---GL-TICV--T-R----I-KGMEKVISEYGG-S--YS---L-C---T-H---LIA-H---IQIVTRKWFQQSIDK--V-CL---SY
NP_729307.1/1-57       NYAMR---GV-VTCF--T-G--I-R-TKLVNLIHSMGG-C--IK---L-T---T-H---LIC-----LTVVRPAWVFAAWAD--N-SL---AT
XP_016865469.1/1-57    EDGTP---KH-IIQM--T-G----F-EALVKLLLKLDCTF--IK---K-C---T-H---LIA-----KWILTKDYIIHSAKS--R-WL---TY
XP_011510659.1/1-55    -PIFL---GC-IICV--T-G----L-KEVQQLTVKHGG-Q--YM---L-C---T-H---LIV-----VHCVTTQWFFDSIEK--F-CQ---IY
NP_524909.1/1-55       -PIFF---GA-NITC--S-G----L-DQVMRLVNDNGG-I--YH---F-V---V-D---IVI-----KDVLLPEWIFDSCNR--Y-AL---DY
NP_524909.1/1-53       ----G---IP-CFSI--S-C----G-AELIARITQLGG-K--VCE--Y-C---T-H---LLC-----KWILNIQYIEQSHAR--D-FL---LY
XP_016861126.1/1-55    -TPLE---DC-VISF--S-Q----C-ESLTFLANLLGA-S--VQ---F-S---T-H---LIL-----LPAVTIAWLLETART--K-RA---HF
NP_172522.2/1-57       EGMFA---GM-VVFM--V-E----I-QIWKQKLVQMGA-V--IE---RVV---T-H---VLA-----ARLMLYQWLEDSLTS--E-KA---LY
NP_001017520.1/1-58    DIKFQ---DL-VVFI--L-EK-K-M-AFLMELARRKGF-R--VE---L-V---T-H---IVA-----PELLDVSWLIECIRA--K-PV---GK
XP_016876061.1/1-56    LSMFR---RH-TVYL--D-S----Y-AIKALELRFHGA-K--VV---L-V---S-H---VII-----FKILKESWVTDSIDK--E-LQ---QY
NP_196956.1/1-57       KSLFK---DL-KFFL--S-R--E-V-ESLQLVITAFGG-M--VS---D-I---T-H---HII-----RVYVQPQWIYDCVNA--I-IL---KY
XP_011533062.1/1-57    GTLFA---DQ-PAMF--V-S--P-A-AKLCELVHLCGG-R--VS---P-A---S-I---VIG-----VKYLSEKWVLVFCGQ--R-ER---TE
NP_609305.1/1-59       RTLFK---GL-KFFI--N-R--E-V-EPLVILIRSFGG-K--VS-DEG-I---T-H---QIV-----RDYIQPQWVFDCVNQ--Q-LL---KY
NP_001019859.1/1-56    --EYE---PL-VACS--S-C----L-TALNQAILQGGF-F--TV---W-C---T-H---LVM-----RPIVKPEYFTEFLKASKK-QP---ES
XP_016878022.1/1-57    KTLFL---GY-AFLL--T-M--A-T-QYTESQLRAGAG-Y--IL---F-Q---C-L---LIA-----IPCVSHVWVHDSCHA--Q-LQ---NY
NP_001185418.1/1-56    DLYLS---DC-RIFL--L-G----F-RKLAKLVRRGGG-S--RY---L-M---T-H---IVV-----IQVVIPSWLEDCDRE--K-EI---NI
NP_524909.1/1-55       -QPLK---GM-TIVV--S-I----Y-DFINATAELLGA-S--VN---F-K---P-L---LVC-----YPVVTSDWLVQCART--Q-KL---GY
XP_011514285.1/1-56    EGFLL---GC-VFAI--A-D----Y-ATWKRIIQAHGG-T--VD---F-C---T-H---LLC-----KRCVTAHWLNTVLKK--K-MV---RA
XP_016876057.1/1-57    SNIFE---DV-EFCV--M-S--G-T-PDLENRIAEFGG-Y--IV---P-T---Y-C---VIA-----HDVVKPAWLLECFKT--S-FV---PR
NP_192120.4/1-55       --GSK---AL-VVCL--T-G----Y-EDIMRMVELMGG-Q--FS---LVV---T-H---LIC-----IKLVNHRWLEDCLKN--K-LL---DY
NP_572907.1/1-58       LNMFR---NL-NAFF--Y-S--P-H-EVAKLLFLQNGG-R--IV---S-L---NLF---ICM-----DKVLNSAWIHQCHRE--I-LL---SF
NP_001185418.1/1-54    --AMD---GL-KVLA--S-G----F-VKIKELVTSMGG-V--LL---A-V---N-F---VIV-----KPIVTLNWLHRCWNE--R-VV---PY
NP_572907.1/1-55       SKLFD---GL-SFCI--L-S----G-HQLQELAVKNGG-C--IV---P-K---C-F---CIA-----CDIVRMEWLLRVC-Q--Q-EL---PR
NP_728695.1/1-58       KTTTG---KI-KVAF--T-M----C-PALETVLKSLKH-VVEIT---P-C---D-L---LVM-----KPVLSTNWLHSVKKT--S-ID---DH
XP_016861126.1/1-56    EGLFS---QK-SFLV--L-G----F-SNIANIIKENAG-K--IM---L-A---D-Y---AVV-----GEVVTNTWLVTCIDY--T-LF---SN
XP_001163122.1/1-58    CELFR---FME-PFYV--S-S--L-C-NNMKELLLGGA-T--LT---R-A---K-Y---IIG-----RVYLDPYWVLDSITN--Q-IQ---KY
XP_011514285.1/1-58    GKPCS---QH-IISV--T-G----F-DDLKLMAYLAGA-K--YT---L-N---T-V---LIC-----IPCVNAQWLGDILLG--FEALR--RY
NP_031375.3/1-57       EEMFR---EV-KYYA--V-G--D-I-PQVIQLLKAGKA-K--EV---N-A---S-H---IIS-----LPVVKPSWVILSVQC--T-LL---GF
NP_001269474.1/1-59    PKLFD---GC-YFYL--W-G--T-F-DNLIKLVTAGGG-Q--IL---K-PRFCT-Q---YII-----VWKAPSSWFIDCVMS--E-LL---S-
NP_650187.2/1-57       PQLFE---GL-VAHF--A-M--E-K-SKLMQQFSQHGG-C--CT---P-A---D-L---VFY-----CRHLNVSWLQSCLDT--S-RQ---LH
XP_005258027.1/1-55    LDIFT---GV-RLYL--P-P--S-T-SRLRRYFVAFDG-D--LV---F-S---T-H---VLG-----AQQVSPEWIWACIRK--R-LV---
NP_001185418.1/1-57    SSVFK---GE-TFCF--S-H--S-F-PQIVEWVNQGGG-E--VV---P-A---H-F---TIE-----TIYVSSHWVRNCLKV--C-LL---SH
XP_016865471.1/1-56    PGAFH---RW-KVVL--L-V----R-DSLIRVLEAGKA-N--VI---K-I---T-H---VIA-----APFYPIQYLGDFLLE--E-IQ---DS
NP_729947.2/1-57       DLFLV---GC-TFYI--V-EYDE-T-PIWLDTIRQFGG-D--IE---Y-V---T-H---VIC-----KRCVTAYWLSDICLK--Q-LM-----
XP_016878022.1/1-57    ENPFQ---NL-KVLL--V-S--D-Q-ELWSEILMTGGA-A--SV---H-F---D-V---VVT-----LPVVSQEWVIQCLIV--E-RI---QH
Q54LN0_DICDI/8/1-56    -GIFN---DF-RIEL--F-T--T-N-DDWSVILLKKCGA-I--IV---M-I---D-A---ILI-----IPILSIFWVIQSILH--R-KL---AH
NP_001318618.1/1-57    DKPFV---GM-MISL--M-G--R-L-QYWKKKIERNGG-K--VS---V-V---T-C---LVV-----LPVVSEAWLIDSVEK--E-AQ---AY
NP_186917.2/1-57       ---LI---PL-VFYLCPS-S----E-QPVQDAVSSIGA-R--IS---L-C---T-H---VLL-----KTIILTNWVMLLAEKCSE-IP---QY
NP_193839.4/1-56       PKLFT---GL-KFYI--M-G--D-F-GYLQDLIVAAGG-T--IL---RPS---T-I---VVF-----ARAASSSWVLDSIAG--Q-IL-----
NP_001318290.1/1-52    -SLAD---HC-MYVD--S-D----I-LKVLKVAGEQGA-K--VI---W-C---A-S---FVV-----KTIVSPLWVLKTVEK--R-Q------
NP_001327947.1/1-55    -GALY---GL-RIVV--Y-G--D-C-DTLKRAVKAGDG-T--IL---A-T---D-F---ALI-----IPCVLSDYLVEYVCK--G--Y---KH
NP_061848.2/1-59       STLFVRGSSM-SFYV--R-P--S---RRLSTLILHGGG-T--VC---Q-G---A-V---LLA-----GDFISTQYILDCVER--E-RL---AY
XP_011513306.1/1-54    -RLLE---GY-EIYV--T-P----G-PQMGEIISCCGG-T--YL---M-Q---R-V---VIT-----LPLLSPEFLLTGVLK--E-A---AF
NP_973758.2/1-56       PKLFE---GL-KFYF--F-G--D-F-EDLQNLVKVAGG-T--IL---E-S---S-I---VVY-----SRLVGHTWVLESIAG--K-LH---G-
NP_009225.1/1-60       RKIFR---GL-EICC--Y-G--P-F-DQLEWMVQLCGA-S--VV---L-F---T-LGPIVVV-----APVVTREWVLDSVAL--Q-CQ---TY
NP_001246702.1/1-53    ---QL---KL-VTTV--S-A----L-QELTKMLEPMGG-T--VT---W-C---S-H---LTM-----KPIVTFPYWRKMLQA--G-WP---DY
NP_728695.1/1-55       -RLLY---GL-HFML--G-K----D-NEMKVIIHSAGG-K--VH---P-D---L-Y---VVV-----VHFIKTEAVMQALVQ--N-IE---EH
NP_001609.2/1-58       DKPLS---NM-KILT--L-G----K-DEVKAMIEKLGG-K--LT---A-A---S-L---CIS-----IRVVSEDFLQDVSAS--K-SL-FLAH
NP_192222.4/1-54       -PLLK---GF-KVCI--T-P----S-GMITDLVKMTQG-Q--VV---S-D---V-L---ILS-----AVIFTSELLLNGIVI--K--L---RF
NP_850165.1/1-57       SERLA---DL-KVSI--A-G--N-T-QPWKKRIEEAGA-E--FH---V-T---S-C---LVV-----VAIVREDYLVDCFKK--R-KL---KY
NP_188785.2/1-54       -PLLQ---GR-RVFI--T-P----N-NTITTLVKAVHG-L--PV---L-E---N-L---LVL-----AEVYSSELLLNGIVT--R-L---RY
NP_001104452.1/1-55    SPPLY---NL-KFSI--I-G--L-K-KELRKRIENLGG-K--FE---I-T---I-A---IIS-----IHIVPIEFLDFVEAD-----E---KY
```
