# Supplementary material for: Exploring the structural landscape of DNA maintenance proteins
Source: Nat Commun. 2024 Sep 5;15:7748. doi: 10.1038/s41467-024-49983-7 (PMC11377751; doi:10.1038/s41467-024-49983-7)
Supplement: Supplementary file 5 — Supplementary Data 2 [file 41467_2024_49983_MOESM5_ESM.pdf]

Supplementary Data 2

SPIDR OB fold domain alignment

```
SPIDR_MOUSE/635-675      WPVCD--RCGNGR-----edGGT----F-S---CGDCS---QL-----VL--S-PLQERH---LHVFLDC
SPIDR_HUMAN/734-774      WPVCD--MCGNGR-----edRGA----F-S---CGDCS---RV-----VT--S-PVLKRH---LQVFLDC
F1MPV0_BOVIN/821-859     WPVCD--LCGSER-------SGT----F-S---CGDCS---HV-----VT--L-PILRRH---LQVFLDS
A0A803K061_XENTR/41-65   CSKCG--KCFSHQkm-----RKP----F-T---CTECN---KG---------------------------
B3DLT7_XENTR/16-41       -HECP--ECGKRFsg-----EKP----F-M---CTKCG---KS-----FR--------------------
E1C5M7_CHICK/285-323     WPTCD--KCGNGK-------SGL----L-Y---CNQCS---EV-----VI--S-PVLKMQ---LEVFLSC
F1N471_BOVIN/653-676     --ECS--KCGKAF-------KKA----Y-A---CTQCQ---KA-----FT--D-----------------
POTE1_MOUSE/505-536      ---------------------HH----Y-G---CKQCS---SLkpalgVV--P-LQYVFV---MVFTLDD
G3N0N0_BOVIN/82-107      ---------HQKV--------GP----Y-E---CSQCG---KF-----F---S-HRFRFL---AHQ----
A0A0C4DG37_HUMAN/49-75   ----------------------P----Y-A---CKECG---KN-----II-yH-SSIQRH---MVVHSGD
D3Z5Z2_MOUSE/341-368     ----------HER-------EKP----Y-K---CDQCN---KA-----F------VYESY---LQVHKKT
A0A803KFK2_XENTR/240-272 --SCT--ECGKHF--tvhtgEKP----F-S---CTECG---KR-----FT-kK-SQL-------------
A0A803JFS6_XENTR/287-313 --GCT--ECGKSF-----tgEKP----Y-A---CTQCP---KS-----FM--D-K---------------
Q49A12_HUMAN/284-311     --KCK--KCGKAF----htgEKP----Y-K---CEKCG---KA-----FN--H-F---------------
A0A803JEM8_XENTR/15-41   QHECP--ECGKRF-------EKP----L-M---CTECG---KS-----FR--R-K---------------
A0A803JFS4_XENTR/81-109  --------------------ERP----F-V---CNECG---KS-----FK-aK-FKLNVH---LRIHTGD
A0A0G2JMG9_HUMAN/202-235 --KCI--ECGKAF--iihtgEKR----Y-K---CEDCG---KA-----FN-rS-SNLT------------
A0A087WPV0_MOUSE/430-458 ----------HER-------ERP----Y-K---CEVCG---KA-----FT-sS-SNLKYH---WRLH---
A6QPD1_BOVIN/392-427     --KCP--DCGQRF--rthtgEKP----Y-Q---CGECG---KS-----FS-rS-SNLATH----------
A0A1D5PPG4_CHICK/228-263 --KCP--ECGKGFtg-----ERP----F-K---CHECG---KT-----FK-sR-SDLSRH---QRI----
A0A803JR47_XENTR/15-48   QHECP--ECGKRF--rvhtgEKP----F-M---CTECG---KN-----FR--R-KQY-------------
A0A803K9Q5_XENTR/326-360 --VCT--ECGKSF------gEKP----F-E---CSECG---KS-----F------RFKRQ---LTLHLQT
A0A803J7H8_XENTR/702-742 WPVCN--ECGSNK-----ekREA----Y-L---CSQCC---QY-----TS--N-PDIRMQ---LEVFLRC
A0A803JX81_XENTR/15-49   QHECP--ECGKRF--rfhtgEKP----F-M---CTECG---KT-----FR--R-KQYL------------
A0A803JV66_XENTR/378-413 --VCT--ECGKSF------gEKP----F-D---CSECG---KS-----FR-vK-SQLNLH---LQTH---
A0A803JFP7_XENTR/201-235 --QCQ--DCGRRF-------ERP----F-Q---CQQCG---KS-----FN-qR-SNYQRH---RLIH---
F1MSU1_BOVIN/294-334     --KCG--ECGKGF--cshtgEKP----Y-Q---CYDCG---KG-----FS-qS-SDLRIH---LRVHT--
F7B8E1_XENTR/102-137     --ACS--ECGKKF-----tgAKP----F-T---CKECG---KS-----FT-cS-SHLSIH---VRT----
F8W7T7_HUMAN/339-377     ---CK--ICGKGFdf-----EKP----Y-E---CKQCG---KL-----LS-hR-SSFRRH---MMAHTGD
A0A3Q1M9Q3_BOVIN/374-414 --RCA--QCGKAF--rihtgEKP----Y-A---CGECG---KT-----FS-rN-TNLTRH---LRVHV--
F1N7R8_BOVIN/465-503     -YKCD--ECGKAF--aihtgEKP----Y-K---CDECG---KA-----FP-vK-STLLRH---QT-----
A0A803JTB2_XENTR/156-194 --LCM--ECGKSFfn---tgEKP----F-S---CTECG---KR-----FT-iK-SQLSDH---YTVH---
F6ZA80_XENTR/255-291     --ICG--KCFSSS-----tgERP----Y-S---CTECG---KR-----FT-qS-SSLVVH---WRVH---
A0A6I8RYV9_XENTR/122-160 --ACT--ECGKSF--kihtgEKP----F-T---CTECG---KS-----FT-eK-SHLRSH---NKI----
A0A3Q4EGA1_MOUSE/297-335 RNRCS--SCGYIV--------------N-T---CTICNqdsSR-----LK--S-FFLSFD---VLVDLTD
A0A3Q1MUX5_BOVIN/617-656 --KCE--ECGKGFkw-----EKP----Y-K---CGECG---KH-----FS-qA-SSLQLH---QSVHTGE
Q3UVL3_MOUSE/195-231     --KCK--ECGKAF--rahsgEKP----Y-E---CKQCG---KT-----F--------RYY---QTFQLHE
F1MBW2_BOVIN/319-358     --ECS--ECGKSF--rvhsgERP----Y-E---CSECG---KS-----FT-fS-SSLRYH---HRVH---
A0A803KBG6_XENTR/17-55   -FKCP--ECGKRYss---tgEKP----F-M---CTECG---KR-----FT-eK-SKLIIH---QRV----
A0A1W2PNY2_HUMAN/95-133  --KCE--ECGKAF--kihtgEKP----Y-V---CEECG---KA-----F------KYSRI---LTTHKRI
A0A3Q1LUH2_BOVIN/165-205 --ECN--ACGKAF--rthltEKP----F-D---CTQCG---NA-----FR--TlSSLKIH---MRVHT--
A0A3Q1N0S8_BOVIN/285-324 --KCT--DCGKSF--rihtgERP----Y-E---CSKCG---KA-----FS-qN-SSLLRH---WRIH---
DDIAS_MOUSE/22-61        YPSCQ--RCFSKI--------KR----F-T---CPKCG---SS-----GDtgS-TNYRYK---LSLKVAE
H3BU10_HUMAN/135-173     RNRCS--SCGYIV--------------N-M---CTTCN---KN-----SL--D-FKSVFLsfhVLIDLTD
A0A087WSP8_MOUSE/470-509 --KCN--ECGKSFtq-----DKP----Y-K---CNDCG---KS-----FT-kS-SNLKVH---HRIHTGD
A0A3Q2U4Z3_CHICK/65-107  QKSCP-wRCSSSVal-----ERP----F-G---CGECG---KS-----FQ-hR-GNLITH---LRVHTGE
A0A0C4DFY4_HUMAN/440-475 --ICS--KCGKAF-----tgEKP----Y-E---CNTCG---KA-----FT--Q----KSH---LNIHQKI
A0A6I8QN04_XENTR/298-337 --TCT--ECGKSF--kihtgEKP----Y-T---CTECG---KS-----FT-vN-SHLVRH---QKIH---
A0A803KB37_XENTR/402-442 --NCT--ECGKGF--kihtgEKP----F-T---CTECG---KS-----FA-qR-YNLVSH---MKIHT--
A0A6I8R766_XENTR/22-59   YPACQ--HCFTRL--------NR----F-E---CPRCG---SQ-----SK--E-AKQRYK---LCLKVAE
A5PJK9_BOVIN/371-410     --VCK--ECGKAFrg-----EKP----Y-E---CFECG---KA-----FR-rT-SHLIVH---QRIHTGE
E1BA02_BOVIN/342-380     RNRCS--VCGYTV--------------S-T---CTTCN---KD-----SS--G-FKSVFLsfdMLIDLTD
E1BQ93_CHICK/211-249     RNRCS--VCRFVV--------------N-T---CTFCG---DI-----SS--D-SKSTFVsfdILVDLTD
A0A3Q1MEJ2_BOVIN/300-342 --KCD--ECGKAF--tvhtgEKP----Y-K---CDECG---KA-----FT-hS-SNLRRH---QKIHTGQ
F6YSY1_XENTR/298-339     ---CT--ECGKGF--kihtgEKP----F-T---CTECG---KG-----FA-hK-KHLVSH---MRIHTGE
A0A7D9NK52_XENTR/333-375 --TCT--ECGKGF--kihtgEKP----Y-T---CTECG---KG-----FA-hK-NNLVRH---LKIHTGE
F1MVJ7_BOVIN/272-314     --KCD--ECGKAF--tvhtgEKR----Y-K---CDECG---KA-----FT-hS-SHLRRH---KKIHTGK
A0A3Q1MSE8_BOVIN/774-816 --ECD--ECGKAF--tvhtgEKP----Y-K---CDECG---KA-----FT-dR-SHLRRH---QKIHTGQ
E1BDX4_BOVIN/445-486     -HKCP--HCDKKF----hiaDGP----L-K---CRECG---KQ-----FT-tS-GNLKRH---LRIHSGE
E5RGX2_HUMAN/16-52       IYICG--ECHTEN-------RDP----I-R---CRECG---YR-----IM-yK-KRTKRC---ILLT---
E1C4L2_CHICK/22-61       YPACQ--SCLSRL--------RR----F-N---CLKCG---CT-----GEaeE-ASYRYR---LSLKIAD
A0A6I8PUK4_XENTR/341-381 RNRCS--RCHYLI--------EL----C-T---YTFCN---EM-----SS--E-PKSVVAcldLSIDLTD
POTE1_CHICK/521-567      KLHCS--KCNTLQ--------GDaikyY-G---CKKCS---TPksvlgIE--P-LQYVFV---MKFTLVD
A0A803JCR1_XENTR/17-60   -FKCP--ECGKRF--kfhtgEEP----F-T---CTECG---KG-----FN-fY-ASLQSH---LRIHTGE
G5E5T7_BOVIN/387-426     AYVCT--ECGKSY-----kgERR----Y-E---CRECG---KS-----FN-fN-SALYYH---KKTHA--
SHLD2_MOUSE/739-778      YAGCA--HCGSEL-------NRI----YrQ---CLSCL---PF-----VG--K-KIFYRP---ALMTIVD
Q0VCV0_BOVIN/478-518     YQACPtqDCNKKV-------NGL----Y-R---CEKCD---SE-----FP--N-FKYRMI---LSVNIAD
A0A6I8Q301_XENTR/453-493 YQACPsqDCNKKV-------NGL----F-R---CEKCD---KE-----FP--N-YKYRLI---LSANIAD
B4DS54_HUMAN/22-61       YPSCQ--KCFSRI--------KR----S-N---CPKCG---ST-----GEsgN-ANYRYK---LSLKVAE
Q6IGE3_DROME/15-52       TYICG--ECHHEN-------RDP----I-R---CRECG---YR-----IM-yK-KRTKRL---VVFDA--
A0A087WUM7_HUMAN/283-324 --KCQ--HCGKAFty---tgEKP----Y-E---CKQCG---KT-----FS-wS-ETLRVH---MRIHTGD
RFA1_HUMAN/478-518       YQACPtqDCNKKV-------NGL----Y-R---CEKCD---TE-----FP--N-FKYRMI---LSVNIAD
RPAB4_BOVIN/16-53        IYICG--ECHTEN-------RDP----I-R---CRECG---YR-----IM-yK-KRTKRL---VVFDA--
RFA1_CHICK/475-515       YQACPsqDCNKKV-------NGL----Y-R---CEKCD---RE-----FP--N-FKYRMM---LLVTIAD
G3XA08_MOUSE/399-443     SYECK--ECGKTF--rshtgEKP----Y-Q---CDQCG---NA-----FA-sS-SYLTTH---LRTHTGE
R4GG09_CHICK/765-804     YSGCA--KCGLEL-------NMI----YkQ---CFRCL---PY-----NK--V-KIFYRP---ALMTVED
RFA1_MOUSE/487-527       YQACPtqDCNKKV-------NGL----Y-R---CEKCD---RE-----FP--N-FKYRMI---LSANIAD
E1BBC8_BOVIN/767-806     YTGCA--KCGLEL-------NKI----YkQ---CFSCL---PF-----TM--K-KIYYRP---ALMTIVD
F1MPR5_BOVIN/710-756     --KCS--DCGKAF--rihtgEKP----Y-I---CTECG---KAfssdfRK--D-FNWKAQ---FSVHQKS
RFA1_DROME/461-501       YRACPqsDCNKKV-------NDQ----F-R---CEKCN---AL-----FP--N-FKYRLL---INMSIGD
SHLD2_HUMAN/696-735      YTGCA--KCGLEL-------NRI----YkQ---CFSCL---PF-----TM--K-KIYYRP---ALMTAID
HDM_DROME/355-397        NRKCI--ACQQHI--------QD----C-A---SDACQ---QYfsdndEP--R-SISYFN---INIHLSD
A8MTK3_HUMAN/248-291     KLHCP--KCHLLQ-------EGD----L-DqdgATKTPd-vKL-----QN--T-SLYDSK---IWTTKNQ
A0A6I8QSC3_XENTR/375-422 KLYCV--KCNSLH-------EDSlrwhY-G---CNRCS---KIksvlgIQ--P-LRYVFI---MKFTFED
E1BGK3_BOVIN/383-426     KLYCP--KCHLLQ-------EID----L-DqegTSKTPd-tTL-----QN--T-SLYNSK---VWTTKDQ
A0A087WQI7_MOUSE/76-117  SYKCI--ECGKSY-----tgDKP----Y-K---CNECG---KY-----FT-kS-SNLKVH---HRLHTGD
A0A6I8SQ60_XENTR/649-688 YSGCE--KCRCEL-------NQV----YeQ---CFFCL---PF-----NQ--V-KTFYRP---ALMTTVS
```
