# Supplementary material for: Exploring the structural landscape of DNA maintenance proteins
Source: Nat Commun. 2024 Sep 5;15:7748. doi: 10.1038/s41467-024-49983-7 (PMC11377751; doi:10.1038/s41467-024-49983-7)
Supplement: Supplementary file 6 — Supplementary Data 3 [file 41467_2024_49983_MOESM6_ESM.pdf]

Supplementary Data 3

M1AP KU core domain alignment

```
M1AP_HUMAN/336-426        lDWDELETNQQHFHALCHSLLKREWLLLAKGEPpsQRIP-TFYVIMPSHS----LTLLVKAVATRELMLPS--TFPL-EDP-HDDSLKNVESMLDSLELE
E1BF42_BOVIN/336-426      lEWDELETNQQHFHALCHSLLKREWLLLAKGEPlkLREP-TFYVILPSHS----PTLLVKAVATRELMLPS--PFPL-EDM-PADSLKTVESMLDDLELE
B1AHC9_HUMAN/321-411      -EESLVIGSSTLFSALLIKCLEKEVAALCRYTP--RRNI-YFVALVPQEEevtpPGFQLVFLPFADDKRKM--PFTE-IMA-TPEQVGKMKAIVEKLRF-
M1AP_MOUSE/336-426        lDWDELETNQQHFHALCHCLLKRDWLLLARGEPlnQSLP-SFYVITPSHS----LTLLVKLVATRELMLPG--FFPL-EDP-PEDSLKIIESTLDSLDLG
D3ZVK0_RAT/336-426        lDWDELETNQQHFHALCHCLMKRDWLLLARGEPlnQSLP-SFYVITPSHS----LTLLVKLVATRELMLPG--FFPL-EDP-PEDSLKIIESTLDSLELG
E9PT85_RAT/369-459        -EESLVNGSSTLFSALLTKCVEKEVIAVCRYTA--RKNV-YFVALVPQEEevtpAGFQLVFLPYADDKRKV--PFTE-VMA-NPEQIDKMKAIVQKLRF-
F1MMD5_BOVIN/373-463      -EESLINGSSTLFSALLTKCLEKEVMAVCRYTP--RRNS-CFVALVPQEEevapPGFQLVFLPYADDKRKV--PFTE-VMA-NPEQVDKMKAIVQKLRF-
A0A0R4J187_MOUSE/369-459 -EESLVSGSSTLFSALLTKCVEKEVIAVCRYTP--RKNV-YFVALVPQEEevtpGGFQLVFLPYADDKRKV--PFTE-VTA-NQEQIDKMKAIVQKLRF-
XRCC6_MOUSE/369-459       -EESLVSGSSTLFSALLTKCVEKKVIAVCRYTP--RKNV-YFVALVPQEEevtpGGFQLVFLPYADDKRKV--PFTE-VTA-NQEQIDKMKAIVQKLRF-
XRCC5_HUMAN/369-457       -DD---EAAAVALSSLIHALDDLDMVAIVRYAY--DKRA-QVGVAFPHIKh-nyECLVYVQLPFMEDLRQY--MFSSkYAP-TEAQLNAVDALIDSMSLA
XRCC5_MOUSE/369-457       -DD---EAAAVALSSLVHALDELNMVAIVRYAY--DKRS-QVGVAFPYIKd-ayECLVYVQLPFMEDLRQY--MFSSkCTP-TEAQLSAIDDLIDSMSLV
G3V817_RAT/369-457        -DD---EAAAVALSSLVHALDELNMVAIVRYAY--DKRA-QVGVAFPFIKd-ayECLVYVQLPFMEDLRQY--MFSSkCTP-TEAQLSAIDDLIDSMSLV
A0A6I8QYR5_XENTR/359-449 -EESLINGSTTLFHALLLRCLAQQVMAICRYTP--RRNT-RFVALVPQDEeskpPGFNLVFLPFADDIRKI--DPPE-ITA-NEEQVDKMKEIVHKLRF-
A7MBA7_BOVIN/369-457      -DD---EAAAVALSSLIHALDELDMVAVVRYVY--NEKT-QVGVAFPLIKd-ayECLVYIQLPFMEDLRQY--MFPSkCTP-TEAQLSAVDALIDSMSLV
A0A6I8SM68_XENTR/387-476 -SDD--EAASVALSSLIHALDEMDMVAIVRYVY--DRRC-QVGVAFPRIKd-kyECLVYIQLPFMEDLRQH--LFSSkFTP-TDSQLSAMDSLIGSMSLV
A0A0R4IB13_DANRE/366-454 -DD---EHAAVALSSLIRALDGLKMAAIVRYAY--DRRS-QVGAAFPCIKk-kyECLLYVQLPYMEDLRQF--TFPMkFMP-SGSQLSAVDALIDSMMLM
F1QZ60_DANRE/368-458      -EEEQISGSSCMFTALLLKCCEKNVFALCKYIP--RRNT-RFVALVPQREeatpPGFHVIYLPFADDIRTV--DPHV-PTA-SDEQVDKMKEIVHKLRF-
A0A0R4IR26_DANRE/100-189 lDWDEMETNNQMFHALCHILKTRDWFLLVRSES--EPGSfSYYVIQSSGS----HSLLLKPVMTRELMLPC--CLPV-EDP-PLLALNTVESSLEQLEKD
A0A6I8SEK8_XENTR/314-407 lDWDTLESNEENFRALCHYLQSQELSLLACTTQ--HSRP-SLGVPVQSHFisdsVALLLRPVAVREIVLPI-ePALP-QPV-KDSTLLKIQDALQPLQVD
A0A2K3D1K3_CHLRE/436-527 -DERSAPGSTAAFIALWRAMLSQDRIAICRLKR--GAS--RHVALVAQDEqseaPGMYLIHLPFADDIRQP--EAVV-NPPlDEAQVAAAAALMQALSLG
KU70_DROME/382-473        -DDQSIIGSKRLFRALWERCLVRDKIAICLFMC--KRKS-RYVALVPVEAplcgDGFKIVYLPEAKHIRHL--DLQDeNTA-DEQKVEFFQKIIKKLRV-
Q9U2D2_CAEEL/389-482      -NDQTTLGSTAIYRTFLDRCWARQQAIVCKYQS--RSKQ-RLMALVPFKKdwlhEGFMLVGQPFREELRDDfkRFEEtEPS-TEEQVNTMKQFVKRLTM-
Q21829_CAEEL/364-456      -LNSPKSGATKATVSLIEAMLSLRVAAICRYTF--HAKS-QLIALLPHQDetgvFYLRSVKLPFSDDMRTL--KFPKlNKP-TVAQLSAVDDLIDCMQLQ
Q9I7M8_DROME/337-430      -HQKHNQSAAVKLDALVRALVSSDRAILCWKIY-sTKFN-QMVVLLPRLAdthpATLYMLEVSYTSQHHFW--DFPAkTEC-SEEQLNAIDQLIDSTDLE
```
