# Supplementary material for: Exploring the structural landscape of DNA maintenance proteins
Source: Nat Commun. 2024 Sep 5;15:7748. doi: 10.1038/s41467-024-49983-7 (PMC11377751; doi:10.1038/s41467-024-49983-7)
Supplement: Supplementary file 7 — Reporting Summary [file 41467_2024_49983_MOESM7_ESM.pdf]

nature portfolio

Corresponding author(s): Kenneth Bødtker schou, Jiri Bartek

Last updated by author(s): 2024-0603

Reporting Summary

Nature Portfolio wishes to improve the reproducibility of the work that we publish. This form provides structure for consistency and transparency in reporting. For further information on Nature Portfolio policies, see our Editorial Policies and the Editorial Policy Checklist.

Statistics

For all statistical analyses, confirm that the following items are present in the figure legend, table legend, main text, or Methods section.

n/a	Confirmed	
☐	☒	The exact sample size (*n*) for each experimental group/condition, given as a discrete number and unit of measurement
☒	☐	A statement on whether measurements were taken from distinct samples or whether the same sample was measured repeatedly
☒	☐	The statistical test(s) used AND whether they are one- or two-sided<br>*Only common tests should be described solely by name; describe more complex techniques in the Methods section.*
☒	☐	A description of all covariates tested
☒	☐	A description of any assumptions or corrections, such as tests of normality and adjustment for multiple comparisons
☒	☐	A full description of the statistical parameters including central tendency (e.g. means) or other basic estimates (e.g. regression coefficient) AND variation (e.g. standard deviation) or associated estimates of uncertainty (e.g. confidence intervals)
☒	☐	For null hypothesis testing, the test statistic (e.g. F, t, r) with confidence intervals, effect sizes, degrees of freedom and P value noted<br>*Give P values as exact values whenever suitable.*
☒	☐	For Bayesian analysis, information on the choice of priors and Markov chain Monte Carlo settings
☒	☐	For hierarchical and complex designs, identification of the appropriate level for tests and full reporting of outcomes
☒	☐	Estimates of effect sizes (e.g. Cohen's *d*, Pearson's *r*), indicating how they were calculated

Our web collection on statistics for biologists contains articles on many of the points above.

Software and code

Policy information about availability of computer code

Data collection	The profile-HMM searches were performed using publicly available databases i.e., the Uniclust30 (UniRef30_2023_02) for HHblits (HHsuite v3.3.0) searches and PfamA (pfamA_35.0) for the HHsearch (HHsuite v3.3.0).The GM gene ontology terms were compiled from the AmiGO 2 database https://doi.org/10.5281/zenodo.10934491. Interactome data for physical GM in interactors were retrieved from the Integrated Interactions Database (IID) version version 2018-05. GM gene co-expression data was retrieved from the CEMiTool Bioconductor package version: Release (3.18) DOI: 10.18129/B9.bioc.CEMiTool.
Data analysis	Sequence remote homology searches were performed with HHsuite v3.3.0, HHpred (Version: 57c8707149031cc9f8edceba362c71a3762bdbf8.<br>Phylogenetic analyses were performed in IQ-Tree v.2.0.<br>. Alignments and ClustalW alignment analysis was performed in<br>Jalview Version: 2.11.3.2. Protein structure analysis was performed in Pymol Version 2.5.5.<br><br>Gene co-expression analysis was performed in CEMiTool Bioconductor package version: Release (3.18) DOI: 10.18129/B9.bioc.CEMiTool. Other than CEMiTool, the filtering and database manipulation was performed using Python version 3.13. GO terms annotation and ontology exploration were performed using the Biopython version 1.83 or goatools version 1.4.4 packages. Database processing was performed using the pandas Version 2.2 package.

For manuscripts utilizing custom algorithms or software that are central to the research but not yet described in published literature, software must be made available to editors and reviewers. We strongly encourage code deposition in a community repository (e.g. GitHub). See the Nature Portfolio guidelines for submitting code & software for further information.

Data

Policy information about availability of data

All manuscripts must include a data availability statement. This statement should provide the following information, where applicable:

- Accession codes, unique identifiers, or web links for publicly available datasets
- A description of any restrictions on data availability
- For clinical datasets or third party data, please ensure that the statement adheres to our policy

Data used in this study i deposited in the Github repository (https://github.com/ELELAB/DDR-candidates.

Accession codes for PDB files are: 6BZG [https://doi.org/10.2210/pdb6BZG/pdb] (yeast SPO16-ZIP2 complex), 1JEY [https://doi.org/10.2210/pdb1JEY/pdb] (human Ku heterodimer bound to DNA), 3FA2 [https://doi.org/10.2210/pdb3FA2/pdb] (human BARD1 Tandem BRCT Domains), 7KK2 [https://doi.org/10.2210/pdb7KK2/pdb] (human PARP1 catalytic domain), 6UEJ [https://doi.org/10.2210/pdb6UEJ/pdb] (human PARP13 bound to RNA), 4B1G [https://doi.org/10.2210/pdb4B1G/pdb] (human PARG catalytic domain), 6I52 [https://doi.org/10.2210/pdb6I52/pdb] (yeast RPA bound to ssDNA), 7SFZ [https://doi.org/10.2210/pdb7SFZ/pdb] (human Mis18a-yippee domain), 6G70 [https://doi.org/10.2210/pdb6G70/pdb] (murine Prpf39), 2LLK [https://doi.org/10.2210/pdb2LLK/pdb] (human DMTF1 Myb (SANT) domain), and 1JJR [https://doi.org/10.2210/pdb1JJR/pdb] (SAP domain of human KU70).

Proteomics data have been deposited in the ProteomeXchange Consortium via the PRIDE partner repository with Project accession: PXD043273.

Research involving human participants, their data, or biological material

Policy information about studies with human participants or human data. See also policy information about sex, gender (identity/presentation), and sexual orientation and race, ethnicity and racism.

Reporting on sex and gender	*Use the terms sex (biological attribute) and gender (shaped by social and cultural circumstances) carefully in order to avoid confusing both terms. Indicate if findings apply to only one sex or gender; describe whether sex and gender were considered in study design; whether sex and/or gender was determined based on self-reporting or assigned and methods used. Provide in the source data disaggregated sex and gender data, where this information has been collected, and if consent has been obtained for sharing of individual-level data; provide overall numbers in this Reporting Summary. Please state if this information has not been collected. Report sex- and gender-based analyses where performed, justify reasons for lack of sex- and gender-based analysis.*
Reporting on race, ethnicity, or other socially relevant groupings	*Please specify the socially constructed or socially relevant categorization variable(s) used in your manuscript and explain why they were used. Please note that such variables should not be used as proxies for other socially constructed/relevant variables (for example, race or ethnicity should not be used as a proxy for socioeconomic status). Provide clear definitions of the relevant terms used, how they were provided (by the participants/respondents, the researchers, or third parties), and the method(s) used to classify people into the different categories (e.g. self-report, census or administrative data, social media data, etc.) Please provide details about how you controlled for confounding variables in your analyses.*
Population characteristics	*Describe the covariate-relevant population characteristics of the human research participants (e.g. age, genotypic information, past and current diagnosis and treatment categories). If you filled out the behavioural & social sciences study design questions and have nothing to add here, write "See above."*
Recruitment	*Describe how participants were recruited. Outline any potential self-selection bias or other biases that may be present and how these are likely to impact results.*
Ethics oversight	*Identify the organization(s) that approved the study protocol.*

Note that full information on the approval of the study protocol must also be provided in the manuscript.

Field-specific reporting

Please select the one below that is the best fit for your research. If you are not sure, read the appropriate sections before making your selection.

☒ Life sciences ☐ Behavioural & social sciences ☐ Ecological, evolutionary & environmental sciences

For a reference copy of the document with all sections, see nature.com/documents/nr-reporting-summary-flat.pdf

Life sciences study design

All studies must disclose on these points even when the disclosure is negative.

Sample size	Results shown in this study were based on two indipendent replicas for each experiment. No sample size deteremination was performed as experiments were performed on cell cultures of roughly 4 million cells per condition subsequently lysed for further analysis. Data used for computational analysis was retrieved form public databases.
Data exclusions	No data was excuded from this study.

Replication	All attempts of replicas were succesfull.
Randomization	Cell cultures used for each experiment were grown in different flasks and at differnet times thus separating cells over time and space.
Blinding	The experiments performed in this study were replicated by two different individualsin the laboratory.

Reporting for specific materials, systems and methods

We require information from authors about some types of materials, experimental systems and methods used in many studies. Here, indicate whether each material, system or method listed is relevant to your study. If you are not sure if a list item applies to your research, read the appropriate section before selecting a response.

Materials & experimental systems

n/a	Involved in the study
☐	☒ Antibodies
☐	☒ Eukaryotic cell lines
☒	☐ Palaeontology and archaeology
☒	☐ Animals and other organisms
☒	☐ Clinical data
☒	☐ Dual use research of concern
☒	☐ Plants

Methods

n/a	Involved in the study
☒	☐ ChIP-seq
☒	☐ Flow cytometry
☒	☐ MRI-based neuroimaging

Antibodies

Antibodies used	Rabbit anti-FLAG (PA1-984B, Invitrogen, RRID: AB_347227, no clone or lot number)<br>Mouse anti-FLAG M2 (F1804, Sigma, no clone or lot number)<br>Mouse anti-GFP (sc-9996, Santa Cruz, clone B-2, no lot number)<br>rabbit anti- RPA2 pSer33 (A300-246A, Bethyl, lot A300-246A-5),<br>rabbit anti-RPA1 (Ab79398, Abcam, clone EPR3472, no lot)<br>rabbit anti-Histone H3 (#9715, Cell Signal, no clone or lot number)<br>Rabbit Phospho-RAD17 (Ser656, ThermoFisher, clone 12HCLC, no lot)
Validation	Rabbit anti-FLAG (PA1-984B): Validation provided at https://www.thermofisher.com/antibody/product/DYKDDDDK-Tag-Antibody-Polyclonal/PA1-984B citing 41 studies confirming its specificity.<br>Mouse anti-FLAG M2 (F1804, Sigma, no clone or lot number). Validation provided https://www.sigmaaldrich.com/DK/en/search/f3165?focus=papers&page=1&perpage=30&sort=relevance&term=F3165&type=citation_search citing 7973 studies confirming its specificity.<br>Rabbit anti-GAPDH (#2118). Validation provided at https://www.cellsignal.com/products/primary-antibodies/gapdh-14c10-rabbit-mab/2118 citing 7799 peer reviewed studies. In addition, showing WB detection of a protein of the right size in humans.<br>Mouse anti-GFP (sc-9996, Santa Cruz, clone B-2, no lot number).Validation provided at https://www.scbt.com/p/gfp-antibody-b-2 citing 8 studies confirming its specificity.<br>Rabbit anti- RPA2 pSer33 (A300-246A, Bethyl, lot A300-246A-5). Validation provided at https://www.fortislife.com/pructs/primary-antibodies/rabbit-anti-phospho-rpa32-s33-antibody/BETHYL-A300-246 citing 192 studies using the antibody.<br>Rabbit anti-RPA1 (Ab79398, Abcam, clone EPR3472, no lot). Validation provided at https://www.abcam.com/products/primary-antibodies/rpa70-antibody-epr3472-ab79398.html citing 65 studies using the antibody.<br>Rabbit anti-Histone H3 (#9715, Cell Signal, no clone or lot number)<br>Rabbit Phospho-RAD17 (Ser656, ThermoFisher, clone 12HCLC, no lot). Validation provided at https://www.cellsignal.com/products/primary-antibodies/histone-h3-antibody/9715 citing 1147 studies using the antibody.

Eukaryotic cell lines

Policy information about cell lines and Sex and Gender in Research

Cell line source(s)	HEK293T: Expressing a mutant variant of SV40 large T antigen (ATCC ACS-4500) from Beckman Coulter Life Sciences.<br>Cell line U-2 OS from ATCC.
Authentication	The cell lines used (U2OS and HEK293T) are purchased from ATCC which guarantees their authentication. Other than that, we have not tested for authentication
Mycoplasma contamination	All cells used in this study were tested negative for mycoplasma.
Commonly misidentified lines (See ICLAC register)	N/A

Plants

Seed stocks	*Report on the source of all seed stocks or other plant material used. If applicable, state the seed stock centre and catalogue number. If plant specimens were collected from the field, describe the collection location, date and sampling procedures.*
Novel plant genotypes	*Describe the methods by which all novel plant genotypes were produced. This includes those generated by transgenic approaches, gene editing, chemical/radiation-based mutagenesis and hybridization. For transgenic lines, describe the transformation method, the number of independent lines analyzed and the generation upon which experiments were performed. For gene-edited lines, describe the editor used, the endogenous sequence targeted for editing, the targeting guide RNA sequence (if applicable) and how the editor was applied.*
Authentication	*Describe any authentication procedures for each seed stock used or novel genotype generated. Describe any experiments used to assess the effect of a mutation and, where applicable, how potential secondary effects (e.g. second site T-DNA insertions, mosiacism, off-target gene editing) were examined.*
